# ‘Do plant-based meats offer a steppingstone towards healthier choices? A cross-sectional audit of the UK market’

**DOI:** 10.1017/jns.2026.10083

**Published:** 2026-03-27

**Authors:** Megan Grace Flint, Simon David Bowles, Jenny Paxman, Anthony Lynn

**Affiliations:** Division of Sustainable Futures and Supply Chain, Sheffield Hallam University - City Campus, UK

**Keywords:** NOVA classification system, Nutrient profiling model, Nutritional composition, Plant-based meat, Meat products

## Abstract

Consumer enthusiasm in plant-based eating has resulted in the rapid expansion of plant-based meat (PBM) products. The extensive processing required to simulate meat warrants further investigation regarding PBMs nutritional quality and healthiness, particularly considering the health halo that has surrounded these products. An online audit of dominant UK supermarkets evaluated PBM (*n* = 209) against ‘standard’ (*n* = 2143) and ‘reduced’ (e.g. low fat) meat equivalents (*n* = 100), across eight product categories. This evaluation included NOVA categorisation, Nutritional Profiling Model (NPM) classification, on-pack claims, micronutrient content and product affordability. PBM products were typically more favourable than ‘standard’ meat equivalents for energy density, dietary fibre, total and saturated fat content. However, they contained significantly higher salt in most product categories. Differences between PBM and ‘reduced’ meat comparators were more nuanced. PBM products were significantly more expensive than ‘standard’ meat equivalents in four of the eight product categories (*p* < .05). Few PBM and zero meat-based products reported micronutrient information. While all PBM and most meat-based products were characterised as ultra-processed, PBM products demonstrated a lower (‘healthier’) NPM score compared to ‘standard’ meat equivalents across all product categories (*p* ≤ .001). Although no significant differences were detected between PBM and ‘reduced’ meat-based products, a greater proportion of PBM products were classified as ‘healthier’ according to NPM compared to ‘standard’ and ‘reduced’ meat equivalents. Thus PBM products may offer healthier alternatives with the potential to synergistically support public and planetary health. Future manufacturing practices should consider cost-effective fortification and reformulation strategies to improve nutritional quality and affordability of PBMs.

## Background

Radical transformation of current food systems is required to provide nutritious food for an estimated global population of 10 billion people by 2050.^(1–3)^ For example, overconsumption of meat, particularly red and processed products, is associated with detrimental health consequences and a reduction in meat and adoption of a plant-based diet will be crucial for future global health as well as climate change targets.^(3–5)^

Approximately half of UK adults have limited their meat consumption^(6)^ and there has been a simultaneous increase in plant-based dietary patterns^(7)^ largely driven by the purported health benefits associated with increased plant consumption.^(8,9)^ Additional concerns regarding the detrimental impact of meat production on the environment and animal welfare has further accelerated this change.^(10,11)^ Rising consumer demand has resulted in rapid expansion of plant-based meat (PBM) products. These are designed to simulate the nutritional and organoleptic properties of their meat-based counterparts and thus offer a steppingstone in the dietary transition away from meat. Between 2018 and 2022, the global PBM industry was reported to have experienced a 302% growth in plant-based products^(12)^ with the UK market share recognised as one of the largest in Europe.^(13)^ Despite recent economic and social challenges^(14)^ the market is projected to increase from £486 to £612 million by 2029.^(6)^

Critical to the adoption of PBM is the product information that enables consumers to make healthy and informed choices. For example, on-pack information provides consumers with details of provenance, shelf-life, preparation and storage information^(15)^ but importantly indicates the nutritional quality of food products.^(16,17)^ In previous studies, consumers have identified product information (including nutritional composition, quality and claims) as a key factor influencing the perceived healthiness of PBM products and subsequent purchasing.^(18,19)^ Nutritional/health claims and other non-claim statements may serve to inflate the so-called health halo surrounding PBM products. For example, products labelled as ‘vegan’ may enhance perceived health and sustainability benefits and thus increase willingness to consume.^(20)^ However, while there is extensive evidence demonstrating numerous health benefits associated with traditional plant-based diets,^(9,21,22)^ this does not infer the same for novel PBM products given the degree of processing required to simulate the nutritional and organoleptic properties of meat-based equivalents. In fact, while subject to ongoing debate, emerging evidence suggests that increased ultra-processed food (UPF) consumption may be associated with an increased risk of adverse health outcomes.^(23–25)^ This may explain the findings of previous published work where consumers have associated PBM products with terms such as ‘unnatural’ and ‘too processed’.^(26–28)^ Unfortunately, there is a paucity of evidence regarding novel PBM products which highlights the need to better understand their healthiness in order to improve consumer literacy and facilitate informed choice.

Previous audit studies have evaluated the nutritional labelling of plant-versus meat-based equivalents in isolation within various market contexts.^(29–36)^ Several studies have also considered degree of processing^(37,38)^ and nutrient profiling^(39–45)^ but few have made a comprehensive evaluation of all these elements together.^(46–48)^ Additional limitations include limited representation of the market share (especially the UK) and failure to adequately consider variation between product categories.

Initial findings suggest that PBM products have a more favourable nutritional profile (e.g. reduced energy density and increased dietary fibre) although concerns have been raised in relation to higher levels of salt and sugar and variations in micronutrients.^(40,49)^ The variation in nutritional composition of PBM underscores the need for a more comprehensive evaluation of the overall nutritional quality of these products. For example, nutritional profiling systems such as the UK Nutritional Profiling Model (NPM) assess the overall healthiness of a product^(50)^ but fail to consider the degree of processing while NOVA classifies products according to their degree of processing independent from their nutritional profile. This complimentary deficit prevents a clearer understanding regarding the association between degree of processing and health value of a product. A better understanding of product healthiness would be gained by considering both dimensions, particularly given the evidence suggesting not all UPF are inherently unhealthy.^(51–53)^ For example, Derbyshire^(51)^ highlighted fifty products characterised as UPF but classified as ‘healthier’ according to the UK NPM.

The rapid expansion of the PBM market over recent years reinforces the need to evaluate the wide range of emerging PBM product categories available across the UK market. To date, the small number of studies in the UK are limited by market representation, inappropriate product categorisation and periods of significant social disruption (e.g. Covid-19 and Brexit). In addition, there has been limited consideration of micronutrient content and affordability which is a key factor in PBM adoption.^(37,39,41,44)^ A recent Irish study with limited representation of key retailers did combine NPM and NOVA classification but ‘data limitations’ prevented the calculation of a complete NPM score and subsequent product classification into ‘healthier’ and ‘less healthy’.^(38)^ The authors also failed to consider product affordability despite recent reports indicating PBM products can be 2–4 times more expensive than their meat-based counterpart.^(54)^

To date, no study has combined both the NOVA and NPM classification systems with product affordability and segmented PBMs by product category to ensure accurate comparisons across a significant proportion of the UK market. Thus, the current study conducted a comprehensive evaluation of plant-versus meat-based products representative of PBM product categories currently available in the UK market. This evaluation included NOVA categorisation, NPM classification, on-pack claims, micronutrient content and product affordability to inform future consumer literacy and promote informed dietary choices.

## Materials and methods

Ethical approval was granted by the Ethics Committee of Sheffield Hallam University (Date 16/02/2023; Reference ER50283699). A cross-sectional audit of dominant UK supermarket websites was conducted between December 2024 and January 2025 to identify PBM and meat-based equivalent products available for purchase. Tesco, Sainsbury’s, Asda, Aldi, Morrisons, Lidl, The Co-operative, Waitrose, Iceland and Ocado were targeted as they collectively represent ∼97% of the total UK grocery market share.^(55)^ However, Lidl was later excluded due to the absence of a product search function on its website. Current literature^(39,40)^ informed the search method, keywords including ‘plant-based’, ‘meat-free’, ‘meat-alternative’, ‘meat-substitute’, ‘vegan’ and ‘vegetarian’ were used to search the nine remaining websites. The current study restricted its scope to novel PBM products designed to simulate the sensory, preparation and consumption experience of their meat-based counterpart. For example, products that offer a direct substitution in familiar dishes (e.g. replacing chicken nuggets with plant-based nuggets or mincemeat with plant-based mince in a Bolognese). Meat-based equivalent products were framed in the context of the definition outlined by the Department for Environment, Food & Rural Affairs and Food Standards Agency^(56)^ ‘“Meat” and similar specific terms like “beef”, “lamb” and “chicken” is mammal or bird skeletal muscle with natural tissue that’s fit for human consumption. Any food or food products derived from plants which were not designed to simulate meat-based products (e.g. tofu, tempeh, falafels and bean burger products) were excluded from the audit (see Supplementary Material 1 for full inclusion criteria). Products sold by multiple retailers were logged once only to avoid duplication.

Eligible products were grouped into one of the following categories: plant-based burgers, sausages, pre-cooked plain ‘chicken’, breaded/battered ‘chicken’, meatballs, mince, bacon and deli meat alternatives (see Supplementary Material 1). A comprehensive search of the selected supermarket websites was conducted to identify suitable meat-based comparators. Within each product category, eligible meat-based products were categorised into two groups for comparative purposes. The first group included ‘standard’ meat-based equivalents. The second group, referred to hereafter as ‘reduced’ meat-based equivalents, included any meat-based equivalent products labelled as reduced fat, low fat, lean or lighter versions in addition to any products designed as ‘healthier’ choices. No meat-based product reported information regarding micronutrient content. Thus, McCance and Widdowson’s Composition of Foods Integrated Database^(57)^ was used to generate reference data for the content of zinc, iron and vitamin B_12_ in meat-based comparators.

Product information including nutritional content (per 100 g), ingredients, price point data (per kg) and any nutritional and/or health claims was extracted from the supermarket websites and recorded. In cases where a nutrient was labelled as ‘trace’ or ‘<’, it was assigned as 0 or rounded to the nearest decimal in line with EU labelling legislation.^(58)^ One product reported an implausible value for vitamin B_12_ which was removed for the analyses. Product eligibility was assessed by the lead author using predefined inclusion/exclusion criteria (supplementary material 1), with ambiguous products resolved though research team discussions. The final database was agreed by the research team.

## Degree of processing

The NOVA classification system was used to assess the degree of processing for each product.^(59)^ NOVA categorises foods into four groups (group 1: unprocessed or minimally processed foods; group 2: processed culinary ingredients; group 3: processed foods; group 4: UPF). Previous authors^(60,61)^ have published a list of markers of UPF underpinned by Monteiro and colleagues^(62)^ descriptive summary of NOVA Group 4. The current study examined the ingredient list for each product against both the NOVA classification definitions^(63)^ and list of UPF markers to determine product categorisation (see Supplementary Material 2).

## Nutritional profile model

The UK Nutrient Profiling Model (NPM) was used to evaluate nutritional quality and ‘health value’ of PBM products against meat-based equivalents. The UK NPM is a recognised tool, scoring a food product according to nutritional content per 100 g.^(64)^ The tool scores each ‘C’ group nutrient (fruit, vegetable and nut content (FVN), dietary fibre and protein) on a scale of 0 to 5 while each ‘A’ group nutrient (energy, saturated fat, total sugar and sodium) is scored on a scale of 0 to 10. Total points attributed to ‘C’ group nutrients are subtracted from total ‘A’ group points to provide a final product score; determining the health value of a product. For example, products scoring equal to or greater than four points would be classified ‘less healthy’ due to a high proportion of nutrients of concern (‘A’ group nutrients). Alternately, those products scoring below four points would be considered ‘healthier’.^(64)^ Similar to Alessandrini and colleagues,^(39)^ examination of the product ingredient list was used to determine any points attributed to the FVN content. Where this indicated FVN contributed more than 40% of the product composition or where four out of the first five ingredients reported were FVN, one positive point would be assigned to the C group for FVN content.^(39)^ Any plant-based protein intended to simulate the animal-based equivalent (e.g. textured pea protein) was not included in the FVN calculation. In cases where the dietary fibre content (required to compute a product’s NPM score) was not reported, we applied the mean dietary fibre content within the plant-or meat-based product category to complete the calculation as described within previous published literature.^(39,65)^

The NOVA categorisation and NPM coding process was undertaken by a Registered Associate Nutritionist (M.F). Similar to previous published work,^(66)^ a second Registered Nutritionist (S.B) conducted a quality control check on 10% of products and there were no conflicts to be resolved.

## Statistical analysis

All statistical analyses were conducted using IBM SPSS Statistics, version 26 (SPSS Inc, Chicago). Shapiro-Wilk tests determined the normality of data distribution in each product category for PBM and their meat-based equivalents. Descriptive statistics were used to measure central tendency, variation and frequency. Most data regarding energy and nutritional content (per 100 g) were not normally distributed and thus presented as median and IQR. In cases where data were normally distributed, the mean and standard deviation were presented.

For product categories with three groups (plant-based, ‘standard’ and ‘reduced’ meat-based products), nutritional composition, NPM score, and price were compared using one-way ANOVA or Kruskal-Wallis tests, dependent on distribution of data. Where appropriate, post-hoc Bonferroni tests were performed. For product categories that did not include ‘reduced’ meat-based products (breaded/battered ‘chicken’ and pre-cooked plain ‘chicken’), Mann-Whitney *U* tests were conducted to determine any significant differences between plant and ‘standard’ meat-based equivalent products. Pearson chi-squared tests were used to explore the association between type of product (plant-based, ‘standard’ and ‘reduced’ meat-based products) and healthiness of products (according to NPM). In all tests, statistical significance was set at *p* < .05.

## Results

A total of 209 PBM products, 2143 ‘standard’ and 100 ‘reduced’ meat-based comparator products were included in the analyses. These were grouped into eight product categories: burgers (*n* = 32 PBM; 98 MB; 12 RMB), sausages (*n* = 30 PBM; 235 MB; 12 RMB), breaded/battered chicken (*n* = 35 PBM; 390 MB), pre-cooked plain chicken (*n* = 44 PBM, 161 MB), meatballs (*n* = 17 PBM; 37 MB; 11 RMB), mince (*n* = 18 PBM; 56 MB; 32 RMB), bacon (*n* = 10 PBM; 268 MB; 21 RMB) and deli meat (*n* = 23 PBM; 898 MB; 12 RMB).

### Nutritional composition

Table 1 presents a comparison of nutritional composition for the eight PBM product categories and their ‘standard’ and ‘reduced’ meat-based comparators. Whilst energy density was significantly lower than ‘standard’ meat-based equivalents for five of the eight product categories considered, the macronutrient composition was variable dependent on category. PBM products across all categories had a significantly higher carbohydrate content, with five and eight categories also demonstrating significantly higher sugar and dietary fibre, respectively, versus ‘standard’ meat-based equivalents (all *p* < .05). For example, plant-based plain ‘chicken’ products contained ten times more dietary fibre than their pre-cooked plain ‘chicken’ comparator (*p* ≤ .001). Conversely, the total fat and saturated fat content of PBM products was significantly lower versus meat equivalents in most product categories. Notably, the reverse was observed for total fat in plant-based breaded/battered and plain ‘chicken’ alternatives (both *p* = .001). In five of the eight product categories, PBMs had a significantly lower protein content (all *p* < .05). Although the Kruskal-Wallis test demonstrated a significant difference in protein content for deli meat products, Bonferroni post-hoc tests revealed no significant difference between the plant-based and meat-based equivalents. Five out of eight PBM categories also demonstrated a significantly higher salt content versus their ‘standard’ meat-based comparators (all *p* < .05). This was particularly notable within the mince category where the salt content of PBM was almost three-fold higher than ‘standard’ meat-based mince products (*p* ≤ .001).

**Table 1.** Comparison of energy density (kcal/ 100g) and nutritional content (g/ 100 g) between plant-based meat (PB) and their ‘standard’ and ‘reduced’ meat-based equivalents (MB and RMB, respectively) within burger, sausage, breaded/battered ‘chicken’, pre-cooked plain ‘chicken’, meatball, mince, bacon and deli meat product categories. Data presented mainly as median and IQR

	Energy (kcal)	Total fat (g)	Saturated fat (g)	Carbohydrate (g)	Total sugar (g)	Protein (g)	Fibre (g)	Salt (g)
**Burgers**								
**PB (** ***n*** **= 32)**	199.50^a^ (43.75)	12.10^a^ (4.40)	2.7^a^ (2.525)	5.60^a^ (2.075)	0.65^a^ (0.475)	*16.00^a^ (4.25)	4.75^a^ (2.90)	1.10^a^ (0.3925)
**MB (** ***n*** **= 98)**	251.50^b^ (37.00)	16.95^b^ (5.00)	7.20^b^ (2.225)	3.15^b^ (2.225)	0.50^b^ (0.30)	*21.00^b^ (4.075)	0.50^b^ (0.40)	0.81^b^ (0.35)
**RMB (** ***n*** **= 12)**	156.00^a^ (37.50)	4.90^a^ (6.25)	2.30^a^ (3.00)	4.85^a^ (2.875)	0.65^ab^ (0.375)	*22.00^b^ (3.575)	0.55^b^ (0.475)	0.82^b^ (0.1575)
***p***	≤.001^2^	≤.001^2^	≤.001^2^	≤.001^2^	.005^2^	≤.001^1^	≤.001^2^	≤.001^2^
**Sausages**								
**PB (** ***n*** **= 30)**	191.50^a^ (65.75)	10.05^a^ (8.725)	1.65^a^ (3.40)	8.35^a^ (6.05)	1.10 (1.35)	13.05^a^ (6.60)	4.30^a^ (2.45)	1.33 (0.305)
**MB (** ***n*** **= 235)**	266.00^b^ (53.00)	19.90^b^ (6.10)	7.30^b^ (2.50)	6.20^b^ (6.50)	1.00 (1.40)	14.80^b^ (3.50)	1.10^b^ (0.75)	1.35 (0.33)
**RMB (** ***n*** **= 12)**	159.00^a^ (59.25)	7.60^a^ (7.10)	3.20^a^ (2.65)	7.30^ab^ (6.45)	1.65 (1.275)	16.55^b^ (1.90)	1.50^b^ (0.70)	1.42 (0.4675)
***p***	≤.001^2^	≤.001^2^	≤.001^2^	.013^2^	.552^2^	.001^2^	≤.001^2^	.627^2^
**Breaded/battered ‘chicken’**								
**PB (** ***n*** **= 35)**	249.00 (59.00)	13.60 (5.50)	1.30 (0.60)	19.00 (6.50)	0.90 (0.80)	11.00 (2.60)	5.70 (2.80)	1.15 (0.38)
**MB (** ***n*** **= 390)**	234.00 (44.25)	11.35 (4.825)	1.60 (1.325)	15.70 (5.80)	0.80 (0.80)	16.00 (3.50)	1.30 (0.80)	0.75 (0.3225)
***p***	.017^3^	.001^3^	.091^3^	≤.001^3^	.418^3^	≤.001^3^	≤.001^3^	≤.001^3^
**Pre-cooked plain ‘chicken’**								
**PB (** ***n*** **= 44)**	176.00 (68.50)	8.45 (5.40)	0.90 (0.775)	3.50 (7.60)	0.50 (0.825)	17.00 (5.85)	5.50 (2.75)	1.10 (0.63)
**MB (** ***n*** **= 161)**	141.00 (69.00)	3.30 (9.25)	0.90 (2.05)	2.30 (4.10)	1.00 (2.15)	23.60 (4.60)	0.50 (0.40)	0.60 (0.37)
***p***	.002^3^	.001^3^	.486^3^	≤.001^3^	.004^3^	≤.001^3^	≤.001^3^	≤.001^3^
**Meatball**								
**PB (** ***n*** **= 17)**	164.00^a^ (52.00)	8.20^a^ (6.45)	1.10^a^ (2.30)	*6.10^a^ (3.25)	1.20^a^ (0.85)	*15.20^a^ (3.50)	5.40^a^ (3.05)	1.00^a^ (0.33)
**MB (** ***n*** **= 37)**	235.00^b^ (41.00)	15.10^b^ (5.05)	6.80^b^ (2.35)	*3.00^b^ (1.90)	0.50^b^ (0.20)	*20.90^**b**^ (4.70)	0.50^b^ (0.10)	0.80^b^ (0.295)
**RMB (** ***n*** **= 11)**	146.00^a^ (36.00)	3.90^a^ (3.20)	1.70^a^ (1.60)	*5.50^a^ (2.90)	0.60^b^ (0.20)	*20.80^b^ (4.10)	0.50^b^ (0.40)	0.90^ab^ (0.21)
***p***	≤.001^2^	≤.001^2^	≤.001^2^	≤.001^1^	≤.001^2^	≤.001^1^	≤.001^2^	.007^2^
**Mince**								
**PB (** ***n*** **= 18)**	168.50^a^ (64.25)	6.80^a^ (7.825)	0.90^a^ (0.95)	6.25^a^ (4.725)	1.25^a^ (2.075)	17.05^a^ (4.575)	5.05^a^ (2.025)	0.7450^a^ (0.3925)
**MB (** ***n*** **= 56)**	210.50^b^ (65.25)	14.50^b^ (8.00)	6.30^b^ (3.975)	0.10^b^ (0.50)	0.10^b^ (0.50)	19.90^a^ (2.325)	0.50^b^ (0.50)	0.20^b^ (0.09)
**RMB (** ***n*** **= 32)**	130.00^c^ (7.75)	4.80^a^ (0.40)	2.00^a^ (0.20)	0.50^b^ (0.50)	0.10^b^ (0.50)	21.90^b^ (2.275)	0.50^b^ (0.50)	0.23^b^ (0.0675)
***p***	≤.001^2^	≤.001^2^	≤.001^2^	≤.001^2^	≤.001^2^	≤.001^2^	≤.001^2^	≤.001^2^
**Bacon**								
**PB**	*179.50^a^ (48.00)	9.15^a^ (10.275)	0.80^a^ (0.90)	5.60^a^ (5.85)	1.35^a^ (1.025)	17.00^a^ (9.425)	5.20^a^ (2.675)	2.365^a^ (0.95)
**MB**	*258.50^b^ (85.25)	17.00^b^ (8.00)	6.30^b^ (3.175)	0.60^b^ (0.50)	0.50^b^ (0.30)	21.05^a^ (10.60)	0.50^b^ (0.50)	3.00^b^ (1.2375)
**RMB**	*164.00^a^ (54.50)	4.60^a^ (2.25)	1.70^a^ (0.70)	1.00^b^ (0.95)	0.50^b^ (0.80)	28.00^b^ (11.00)	0.50^b^ (0.10)	2.80^ab^ (0.555)
***p***	≤.001^1^	≤.001^2^	≤.001^2^	≤.001^2^	≤.001^2^	.001^2^	≤.001^2^	.010^2^
**Deli meat**								
**PB**	212.00^a^ (103.00)	9.70 (5.80)	0.80^a^ (0.60)	5.90^a^ (5.6)	1.40^a^ (3.00)	19.5 (12.00)	4.45^a^ (3.525)	1.80 (0.71)
**MB**	140.50^a^ (170.00)	4.90 (18.95)	1.70^b^ (7.525)	1.00^b^ (1.70)	0.60^b^ (0.70)	22.50 (5.50)	0.50^b^ (0.30)	2.00 (1.7875)
**RMB**	109.00^b^ (116.50)	2.75 (12.35)	0.95^ab^ (5.025)	1.95^b^ (1.95)	1.40^ab^ (1.3)	18.20 (6.675)	0.50^b^ (0.50)	2.1750 (0.5475)
***p***	.023^2^	.351^2^	.003^2^	≤.001^2^	≤.001^2^	.013^2^	≤.001^2^	.625^2^

IQR, Interquartile range.

1 One-Way ANOVA test for differences between three comparative groups (PB, MB and RMB).

2 Kruskal-Wallis test for differences between three comparative groups (PB, MB and RMB).

3 Mann-Whitney U tests for differences between two comparative groups (PB and MB).

^abc^Different letters within each column denote significant differences in pairwise comparison (*p* < .05).

* Data are normally distributed and thus presented as mean and standard deviation.

Comparison between PBM and the six ‘reduced’ meat-based equivalents product categories was more nuanced (Table 1). Only two categories identified a significant difference in energy density where PBM products were greater than ‘reduced’ meat comparators. While no category observed a significant difference in total and saturated fat content, five of the six categories identified a significantly lower protein content in PBM versus ‘reduced’ meat-based equivalents (*p* ≤ .001). Carbohydrate content, particularly sugar, was variable dependent on product category. However, dietary fibre was consistently higher in PBM versus ‘reduced’ meat-based equivalents across all product categories (all *p* ≤ .001). The most notable difference being that plant-based meatballs were almost ten times greater in dietary fibre versus the ‘reduced’ meatball products. Only the burger and mince category demonstrated PBM products to be comparatively higher in salt content (both *p* ≤ .001).

Vitamin B_12_ and iron were the most commonly reported micronutrients across all PBM product categories, followed by zinc (present on 17.7%, 17.7% and 4.8% of PBM products, respectively; Table 2). Conversely, no meat-based product provided data regarding micronutrient content. Therefore, McCance and Widdowson’s Composition of Foods Integrated Database^(67)^ was used to generate reference data for the content of zinc, iron and vitamin B_12_ for meat-based comparators (Table 2). Table 2 also presents the UK dietary reference values (DRVs) for comparison. Across all categories, PBM products demonstrated a relatively higher percentage contribution towards the UK Lower Nutrient Reference Intake (LRNI) and Reference Nutrient Intake (RNI) for iron and zinc compared to the meat reference data. However, the percentage contribution from meat-based reference data towards UK LRNI and RNI for vitamin B_12_ was relatively higher than PBM across three of the four categories compared.

**Table 2.** Frequency (number and %) of products reporting content of micronutrients and the median and range content (per 100 g) across plant-based meat product categories alongside reference data for meat-based equivalents (per 100 g). UK Dietary Reference Values including Lower Reference Nutrient Intake and Reference Nutrient Intake are also presented for Males (M) and Females (F)

	Burgers			Sausages			Breaded/ battered ‘chicken’			Pre-cooked plain ‘chicken’			Meatballs		
	*N* (%)	PB*	MB**	*N* (%)	PB*	MB**	*N* (%)	PB*	MB**	*N* (%)	PB*	MB**	*N* (%)	PB*	MB**
**Iron (mg)**	3−9.4	4.9 (3.70–11.20)	2.1	3−10	2.8 (2.40–7.40)	0.9	4−11.4	3.37 (2.30–4.48)	0.65	15−34.1	3.3 (2.30–4.60)	0.4	3−17.6	4.1 (2.30–4.60)	NA
**Zinc (mg)**	2−6.3	2.95 (1.70–4.20)	2.5	2−6.7	3.25 (1.80–4.70)	0.9	2−5.7	2.63 (2.41–2.85)	0.6	1−2.3	4.00 (4.00–4.00)	0.8	1−5.9	2.8 (2.80–2.80)	NA
**B**_**12**_ **(μg)**	3−9.4	1.70 (1.00–3.00)	2	2−6.7	1.59 (0.28–2.90)	1	4 (11.4)	0.71 (0.59–1.40)	2.8	16−36.4	0.58 (0.40–1.00)	Tr	2−11.8	0.56 (0.41–0.70)	NA

	Mince			Bacon			Deli meat			UK dietary reference values***			
										LRNI		RNI	
	*N* (%)	PB*	MB**	*N* (%)	PB*	MB**	*N* (%)	PB*	MB**	M	F	M	F
**Iron (mg)**	3−16.7	4.9 (4.90–5.00)	1.4	4−40	3.8 (1.90–5.30)	0.4	2−8.7	5.59 (5.40–5.78)	NA	4.7	8	8.7	14.8♦
**Zinc (mg)**	1−5.6	4.2 (4.20–4.20)	3.9	0 0	NA	1.2	1−4.3	3.84 (3.84–3.84)	NA	5.5	4	9.5	7
**B**_**12**_ **(μg)**	4−22.2	1 (0.71–3.00)	2	4−40	0.68 (0.30–0.78)	Tr	2−8.7	1.57 (1.13–2.00)	NA	1	1	1.5	1.5

PB, Plant-based; MB, Meat-based; LRNI, Lower reference nutrient intake; RNI, Reference nutrient intake; M, male; F, female; NA, data not available; Tr, trace.

* Micronutrient data provided on PBM product packaging presented as median (range) per 100 g serving.

** Reference data generated by McCance and Widdowson’s Composition of Foods Integrated Dataset 2021 (57).

*** UK Dietary Reference Values^(113,114)^ presented in mg or μg per day for adults aged 19–50 y (LRNI) and 19–64y (RNI).

♦ RNI presented for UK females aged 19–50y.

### Degree of processing

**Figure 1.**
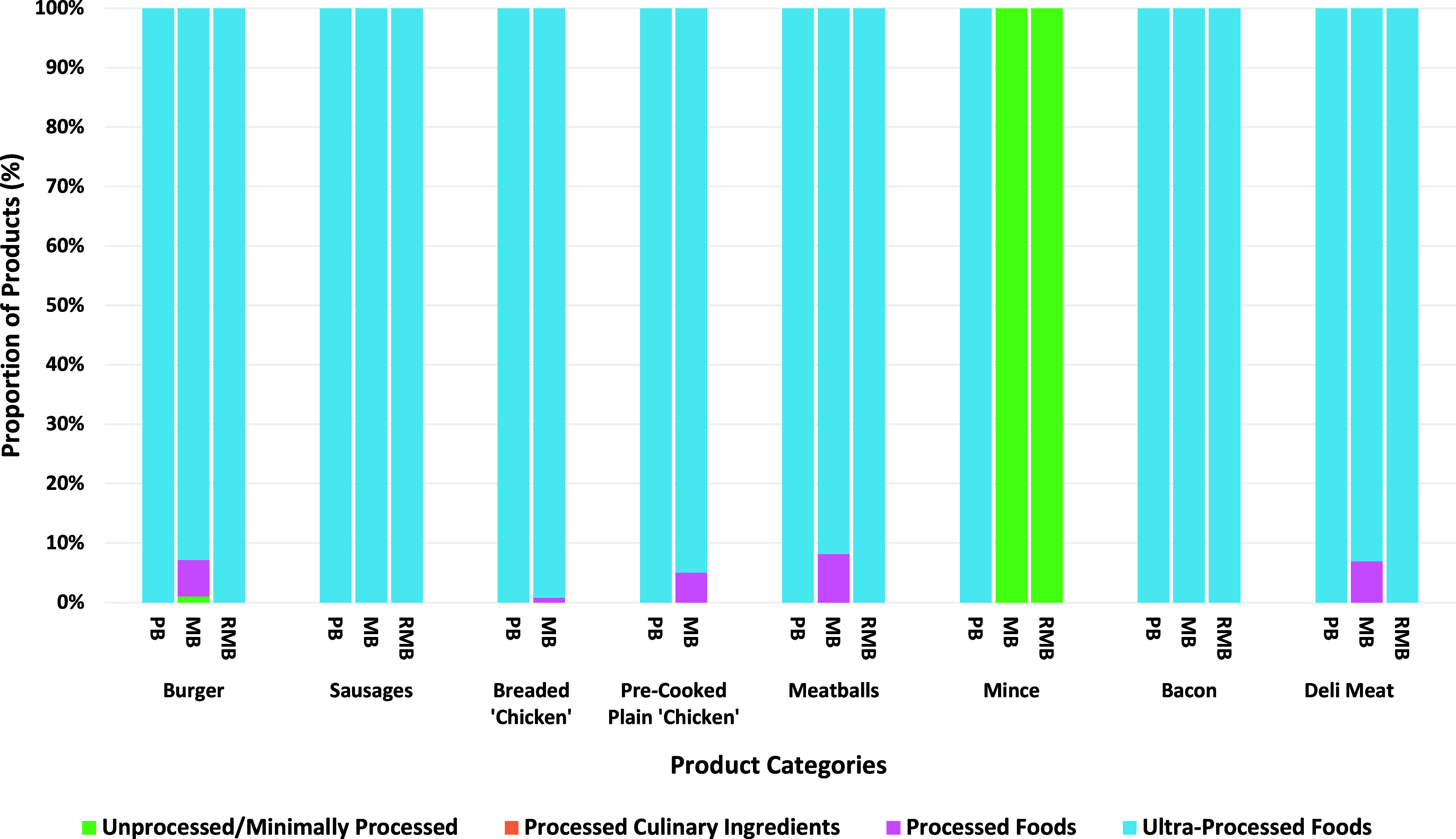
Proportion of PB, MB and RMB products categorised as NOVA Group 1 (Unprocessed/Minimally Processed); Group 2 (Processed Culinary Ingredients); Group 3 (Processed Foods) or Group 4 (Ultra-Processed Foods) according to the NOVA classification system across each product category. PB, Plant-based; MB; Meat-based; RMB; ‘Reduced’ meat-based.

All PBM product categories and all-but one ‘reduced’ meat-based product categories were characterised as UPFs according to NOVA (Figure 1). The vast majority of ‘standard’ meat-based products were also characterised as ultra-processed with the exception of a small proportion of products from the burger, breaded/battered chicken, pre-cooked plain chicken, meatball and deli meat product categories which were assigned to the processed food group. Only the ‘standard’ and ‘reduced’ meat-based mince products were classified as unprocessed/minimally processed foods along with just one ‘standard’ meat-based product from the burger category.

### Nutrient profiling model

All PBM products demonstrated a significantly lower (‘healthier’) NPM score versus ‘standard’ meat-based equivalents (all *p* ≤ .001; Table 3). A notable example being within the meatball category where the average difference between the plant-based and ‘standard’ meat-based equivalent products was 15 NPM points (−3.00 (−5.25) vs 12 (5.00), *p* ≤ .001). Chi-squared test revealed a significant association between type of product (plant-based, ‘standard’ meat-based or ‘reduced’ meat-based) and healthiness according to the NPM (*X* ^2^(2) = 137.115, *p* < .001). A total of 79.4% of PBM products were classified as ‘healthier’ versus 57% and 38.7% of ‘reduced’ meat-based and ‘standard’ meat-based equivalent products, respectively.

**Table 3.** Comparison of nutrient profiling model scores between plant-based meat and their meat-based equivalents within burger, sausage breaded/battered ‘chicken’, plain ‘chicken’, meatball, mince, bacon and deli meat product categories. Data presented as median and interquartile range

	Burgers	*p*	Sausages	*p*	Breaded/ battered ‘chicken’	*p*	Pre-cooked plain ‘chicken’	*p*	Meatballs	*p*	Mince	*p*	Bacon	*p*	Deli meat	*p*
**PB**	0.00^a^ (−1.00)	≤.001^1^	1.00^a^ (5.75)	≤.001^1^	−2.00^a^ (−2.00)	≤.001^2^	−3.00^a^ (−5.25)	≤.001^2^	−3.00^a^ (−3.00)	≤.001^1^	−4.00^a^ (−6.50)	≤.001^1^	7.00^a^ (2.25)	≤.001^1^	2.00^a^ (7.00)	.001^1^
**MB**	12.00^b^ (2.25)		15.00^b^ (4.00)		1.00^b^ (1.75)		−1.00^b^ (−1.00)		12.00^b^ (5.00)		3.00^b^ (9.75)		19.00^b^ (4.00)		11.00^b^ (16.00)	
**RMB**	1.00^a^ (2.75)		6.00^a^ (8.00)		–		–		1.00^a^ (2.00)		−2.00^a^ (−4.00)		13.00^a^ (1.00)		5.00^ab^ (10.00)	

PB, Plant-based; MB, Meat-based; RMB, ‘Reduced’ meat-based.

1 Kruskal-Wallis test for differences between three comparative groups (PB, MB and RMB).

2 Mann-Whitney *U* tests for differences between two comparative groups (PB and MB).

^ab^Different letters within each column denote significant differences in pairwise comparison (*p* < .05).

### Product claims

#### Nutritional and health claims

Nutritional claims were present on over three-quarters (77%) of PBM products surveyed. Conversely, fewer ‘standard’ and ‘reduced’ meat-based equivalent products made nutritional claims (11.2% and 43%, respectively) (Table 4). Nutritional claims related to the protein (‘source of’ or ‘high in’), saturated fat (‘low’ or ‘reduced’) and dietary fibre content (‘source of’ or ‘high in’) were the most presented claims for PBM products (71.3%, 36.8% and 26.3%, respectively). For meat-based equivalents, claims regarding protein content were most common within ‘standard’ comparators while claims regarding fat content were most common for ‘reduced’ comparators (10.3% and 33.0%, respectively). Very few products made claims regarding reduced salt (0%, .2% and 2% of PBM, ‘standard’ meat-based and ‘reduced’ meat-based equivalents, respectively) and only one claim was made for ‘reduced energy’ by a ‘reduced’ meat-based comparator.

**Table 4.** Frequency (number and %) of nutritional claims present on plant-based meat and their standard and ‘reduced’ meat-based equivalent products across each product category

	Nutritional claims															
	Protein		Dietary fibre		Total fat		Saturated fat		Sugar		Iron		Vitamin B_12_		Reduced energy	Reduced salt
	Source of	High in	Source of	High in	Low	Reduced	Low	Reduced	Low	No added	Source of	High in	Source of	High in		
**PB burger**	1 (3.1)	16 (50.0)	7 (21.9)	2 (6.3)	0 (0.0)	0 (0.0)	5 (15.6)	4 (12.5)	0 (0.0)	0 (0.0)	0 (0.0)	1 (3.1)	0 (0.0)	1 (3.1)	0 (0.0)	0 (0.0)
**MB burger**	0 (0.0)	1 (1.0)	0 (0.0)	0 (0.0)	0 (0.0)	0 (0.0)	0 (0.0)	0 (0.0)	0 (0.0)	0 (0.0)	0 (0.0)	0 (0.0)	0 (0.0)	0 (0.0)	0 (0.0)	0 (0.0)
**RMB burger**	0 (0.0)	1 (8.3)	0 (0.0)	0 (0.0)	0 (0.0)	3 (25.0)	0 (0.0)	0 (0.0)	0 (0.0)	0 (0.0)	0 (0.0)	0 (0.0)	0 (0.0)	0 (0.0)	0 (0.0)	0 (0.0)
**PB sausage**	1 (3.3)	21 (70.0)	7 (23.3)	2 (6.7)	1 (3.3)	0 (0.0)	1 (3.3)	6 (20.0)	0 (0.0)	0 (0.0)	0 (0.0)	0 (0.0)	0 (0.0)	0 (0.0)	0 (0.0)	0 (0.0)
**MB sausage**	1 (.4)	3 (1.3)	0 (0.0)	0 (0.0)	0 (0.0)	0 (0.0)	0 (0.0)	0.00	2 (0.9)	0 (0.0)	0 (0.0)	0 (0.0)	0 (0.0)	0 (0.0)	0 (0.0)	0 (0.0)
**RMB sausage**	0 (0.0)	1 (8.3)	0 (0.0)	0 (0.0)	1 (8.3)	9 (75.0)	0 (0.0)	0.00	0 (0.0)	0 (0.0)	0 (0.0)	0 (0.0)	0 (0.0)	0 (0.0)	0 (0.0)	0 (0.0)
**PB breaded/ battered chicken**	14 (40.0)	10 (28.6)	6 (17.1)	8 (22.9)	0 (0.0)	0 (0.0)	4 (11.4)	0.00	0 (0.0)	0 (0.0)	2 (5.7)	0 (0.0)	4 (11.4)	0 (0.0)	0 (0.0)	0 (0.0)
**MB breaded/ battered chicken**	4 (1.0)	20 (5.1)	2 (0.5)	0 (0.0)	0 (0.0)	0 (0.0)	0 (0.0)	0.00	0 (0.0)	1 (0.3)	0 (0.0)	0 (0.0)	0 (0.0)	0 (0.0)	0 (0.0)	0 (0.0)
**RMB breaded/ Battered chicken**	–	–	–	–	–	–	–	–	–	–	–	–	–	–	–	–
**PB plain chicken**	1 (2.3)	30 (68.2)	10 (22.7)	11 (25.0)	0 (0.0)	0 (0.0)	12 (27.3)	0 (0.0)	0 (0.0)	0 (0.0)	9 (20.5)	0 (0.0)	9 (20.5)	0 (0.0)	0 (0.0)	0 (0.0)
**MB pre-cooked plain chicken**	0 (0.0)	91 (56.5)	0 (0.0)	0 (0.0)	6 (3.7)	0 (0.0)	0 (0.0)	0 (0.0)	0 (0.0)	0 (0.0)	0 (0.0)	0 (0.0)	0 (0.0)	0 (0.0)	0 (0.0)	0 (0.0)
**RMB pre-cooked plain chicken**	–	–	–	–	–	–	–	–	–	–	–	–	–	–	–	–
**PB meatballs**	0 (0.0)	13 (76.5)	2 (11.8)	5 (29.4)	0 (0.0)	0 (0.0)	2 (11.8)	1 (5.9)	0 (0.0)	0 (0.0)	2 (11.8)	0 (0.0)	2 (11.8)	0 (0.0)	0 (0.0)	0 (0.0)
**MB meatballs**	0 (0.0)	1 (2.7)	0 (0.0)	0 (0.0)	0 (0.0)	0 (0.0)	0 (0.0)	0 (0.0)	0 (0.0)	0 (0.0)	0 (0.0)	0 (0.0)	0 (0.0)	0 (0.0)	0 (0.0)	0 (0.0)
**RMB meatballs**	0 (0.0)	0 (0.0)	0 (0.0)	0 (0.0)	0 (0.0)	1 (9.1)	0 (0.0)	0 (0.0)	0 (0.0)	0 (0.0)	0 (0.0)	0 (0.0)	0 (0.0)	0 (0.0)	1 (9.1)	0 (0.0)
**PB mince**	0 (0.0)	14 (77.8)	5 (27.8)	2 (11.1)	0 (0.0)	1 (5.6)	8 (44.4)	1 (5.6)	0 (0.0)	0 (0.0)	1 (5.6)	0 (0.0)	1 (5.6)	0 (0.0)	0 (0.0)	0 (0.0)
**MB mince**	0 (0.0)	0 (0.0)	0 (0.0)	0 (0.0)	0 (0.0)	0 (0.0)	0 (0.0)	0 (0.0)	0 (0.0)	0 (0.0)	0 (0.0)	0 (0.0)	0 (0.0)	0 (0.0)	0 (0.0)	0 (0.0)
**RMB mince**	0 (0.0)	8 (25.0)	0 (0.0)	0 (0.0)	0 (0.0)	1 (3.1)	0 (0.0)	0 (0.0)	0 (0.0)	0 (0.0)	0 (0.0)	0 (0.0)	0 (0.0)	0 (0.0)	0 (0.0)	0 (0.0)
**PB bacon**	0 (0.0)	8 (80.0)	2 (20.0)	0 (0.0)	2 (20.0)	0 (0.0)	0 (0.0)	1 (10.0)	0 (0.0)	0 (0.0)	2 (20.0)	0 (0.0)	2 (20.0)	0 (0.0)	0 (0.0)	0 (0.0)
**MB bacon**	0 (0.0)	5 (1.9)	0 (0.0)	0 (0.0)	0 (0.0)	2 (0.7)	0 (0.0)	0 (0.0)	0 (0.0)	0 (0.0)	0 (0.0)	0 (0.0)	0 (0.0)	0 (0.0)	0 (0.0)	4 (1.5)
**RMB bacon**	0 (0.0)	0 (0.0)	0 (0.0)	0 (0.0)	0 (0.0)	11 (52.4)	0 (0.0)	0 (0.0)	0 (0.0)	0 (0.0)	0 (0.0)	0 (0.0)	0 (0.0)	0 (0.0)	0 (0.0)	2 (9.5)
**PB deli meat**	1 (4.3)	19 (82.6)	7 (30.4)	1 (4.3)	0 (0.0)	0 (0.0)	10 (43.5)	0 (0.0)	0 (0.0)	0 (0.0)	0 (0.0)	2 (8.7)	0 (0.0)	2 (8.7)	0 (0.0)	0 (0.0)
**MB deli meat**	26 (2.9)	69 (7.7)	0 (0.0)	0 (0.0)	3 (0.3)	2 (0.2)	0 (0.0)	0 (0.0)	1 (0.1)	0 (0.0)	0 (0.0)	0 (0.0)	0 (0.0)	0 (0.0)	0 (0.0)	1 (0.1)
**RMB deli meat**	2 (16.7)	0 (0.0)	0 (0.0)	0 (0.0)	3 (25.0)	4 (33.3)	0 (0.0)	0 (0.0)	0 (0.0)	0 (0.0)	0 (0.0)	0 (0.0)	0 (0.0)	0 (0.0)	0 (0.0)	0 (0.0)
**Total PB**	18 (8.6)	131 (62.7)	46 (22.0)	31 (14.8)	3 (1.4)	1 (0.5)	42 (20.1)	13 (6.2)	0 (0.0)	0 (0.0)	16 (7.7)	3 (1.4)	18 (8.6)	3 (1.4)	0 (0.0)	0 (0.0)
**Total MB**	31 (1.4)	190 (8.9)	2 (0.1)	0 (0.0)	9 (0.4)	4 (0.2)	0 (0.0)	0 (0.0)	3 (0.1)	1 (0.0)	0 (0.0)	0 (0.0)	0 (0.0)	1 (0.0)	0 (0.0)	5 (0.2)
**Total RMB**	2 (2.0)	10 (10.0)	0 (0.0)	0 (0.0)	4 (4.0)	29 (29.0)	0 (0.0)	0 (0.0)	0 (0.0)	0 (0.0)	0 (0.0)	0 (0.0)	0 (0.0)	0 (0.0)	1 (1.0)	2 (2.0)

There were numerous products which did not present certain nutritional claims despite meeting the eligibility criteria outlined by the European Commission^(58)^ (see Supplementary Material 3). For example, although 85.2% of PBM products were eligible to claim ‘source of fibre’, and 31.1% met the criteria for ‘high fibre’, these claims were only made by 22% and 14.8% of PBM products, respectively. In addition, while almost all ‘standard’ meat-based products complied with the criteria for ‘source of protein’, fewer than 2% of products presented this claim. For example, all ‘standard’ meat-based mince products met the criteria to claim both ‘source of protein’ and ‘high protein’, but no products made these claims. Although most PBM and meat-based products were eligible to claim low sugar, no PBM products, and very few meat-based products (.1%) made a claim regarding sugar content. Notably, only one meat-based product made a claim regarding micronutrient content. Similar to the proportion of PBMs presenting micronutrient data (Table 2), approximately one in ten PBM products made a claim regarding iron and vitamin B_12_.

Across all PBM categories, only one product (0.5%) made a protein-related health claim. Similarly, a small proportion of ‘standard’ and ‘reduced’ meat-based equivalents (1.9% and 7%, respectively) made health claims related to protein or gut function.

### Additional statements

Almost all (97.1%) PBM products made at least one additional statement (see Supplementary Material 4). The most common being any statement related to the plant-based composition and suitability to vegan and vegetarian consumers which were present on 96.7% of PBM products. Other common statements on PBM, also present on ‘standard’ and ‘reduced’ meat-based equivalents, included statements related to natural ingredients and assuring the absence of artificial ingredients (11%, 13.3% and 7%, respectively) and gluten-free claims (8.6%, 15.2% and 25%, respectively).

### Product cost

Table 5 highlights the difference in cost per kilogram of PBM and their meat-based comparators across each product category. Plant-based burgers, sausages, breaded/battered ‘chicken’ and bacon products were significantly more expensive versus their ‘standard’ meat-based equivalent with median prices +31.2%, +22.5%, +46.9% and +56.2%, respectively (all *p* < .05). Plant-based sausage and bacon products were also found to be significantly more expensive in contrast to their ‘reduced’ meat-based counterpart with plant-based bacon priced over two-fold higher than ‘reduced’ equivalents. Conversely, no significant differences in price were observed between plant-based plain ‘chicken’, meatball, mince and deli meat products and their meat-based equivalent products. However, ‘reduced’ mince products were highlighted as significantly more expensive (+38.5%) compared to ‘standard’ mince products.

**Table 5.** Comparison of price difference (£ per kg) between plant-based meat and their meat-based equivalents within burger, sausage, breaded/battered ‘chicken’, plain ‘chicken’, meatball, mince, bacon and deli meat product categories. Data presented as median and interquartile range

	Burgers	*P*	Sausages	*p*	Breaded/ battered ‘chicken’	*p*	Pre-cooked plain ‘chicken’	*p*	Meatballs	*p*	Mince	*p*	Bacon	*p*	Deli Meat	*p*
**PB**	£11.56^a^ (7.32)	.012^1^	£9.19^a^ (4.73)	.001^1^	£12.85^a^ (5.62)	≤.001^2^	£13.07 (8.74)	.498^2^	£10.70 (7.48)	.900^1^	£8.17^ab^ (6.08)	.003^1^	£22.77^a^ (10.65)	.001^1^	£25.00 (11.37)	.053^1^
**MB**	£8.81^b^ (3.79)		£7.50^b^ (4.25)		£8.75^b^ (5.36)		£12.40 (8.81)		£11.03 (6.95)		£7.22^a^ (3.89)		£14.58^b^ (9.80)		£18.80 (13.77)	
**RMB**	£8.54^ab^ (5.07)		£5.79^b^ (4.56)		–		–		£9.56 (3.71)		£10.00^b^ (3.39)		£10.97^b^ (5.86)		£15.40 (6.90)	

PB, Plant-based; MB, Meat-based; RMB, ‘Reduced’ meat-based.

1 Kruskal-Wallis test for differences between three comparative groups (PB, MB and RMB).

2 Mann-Whitney U tests for differences between two comparative groups (PB and MB).

^ab^Different letters within each column denote significant differences in pairwise comparison (*p* < .05).

## Discussion

This comprehensive evaluation of PBM versus meat-based comparator products representative of current UK market product categories is the first study to simultaneously evaluate healthiness and degree of processing alongside micronutrient content, on-pack claims and affordability. Despite between product category heterogeneity, our findings highlight lower energy density, total fat and saturated fat but higher dietary fibre in PBM compared to ‘standard’ meat-based equivalents. We also identify considerations for future PBM development (e.g. reduced salt content). PBM and ‘reduced’ meat-based products demonstrated significantly lower (‘healthier’) NPM scores compared to ‘standard’ meat-based equivalents across all product categories. Although no difference was found between ‘reduced’ meat-based and PBM products, a greater proportion of PBMs were classified as ‘healthier’. Notably, all PBM, all-but one ‘reduced’ meat-based products and most ‘standard’ meat-based products were categorised as UPF. Hence, while ‘reduced’ meat-based products offer a ‘healthier’ alternative to ‘standard’ meat-based products, there may be incremental health benefits when adopting PBM, that may offer a steppingstone towards healthier, more sustainable food systems.

### Nutritional composition

Energy density was significantly lower in PBM versus their ‘standard’ meat-based equivalents across most categories, reflecting the findings of previous studies conducted within the UK^(37,39)^ and across Europe,^(34,35,40)^ Australia^(40)^ and Asia.^(45,68,69)^ As excess energy intake is a key factor in global obesity,^(70,71)^ transitioning from meat to PBM may support weight loss and/or management.^(72)^ The lower energy density of PBM observed in the burger, sausage, mince, meatball and bacon categories may be attributed to their lower total fat content. The comparatively higher energy density and total fat observed in breaded/battered and plain ‘chicken’ aligns with previous work in the UK and data from European and Malaysian markets.^(32,38,41,45)^ Over three-quarters of UK adults currently exceed the recommended intake for saturated fats^(73)^ and the association of adverse health consequences with high saturated fat consumption (e.g. cardiovascular disease) are widely reported.^(74,75)^ As previously reported,^(33,34,37–40,43)^ PBM products were lower in saturated fat compared to ‘standard’ and ‘reduced’ meat equivalents across most categories. Meat contributes significantly to saturated fat consumption in the UK^(73)^ and substituting meat products with PBM may reduce saturated fat intake and facilitate improved health.^(76–78)^

Significantly higher carbohydrate content in plant-versus ‘standard’ meat-based equivalents was observed across all product categories. Previous studies both within and outside of the UK have reported similar findings.^(33,34,36,38,40,43,79)^ Although replicated in fewer product categories for ‘reduced’ meat-based products, it suggests that transition to PBM from meat-based products may increase carbohydrate intake. This could be due to high carbohydrate content of legumes and cereals, key constituents of PBM, or carbohydrate-rich additives in PBM to simulate the organoleptic properties of fat.^(80,81)^

Carbohydrate is comprised of different components, including total sugar content. Comparatively higher sugar was found in some PBM products but was inconsistent across categories, again reflecting previous studies.^(33–36,38,40,43,48,68,69,79)^ Interestingly, plant-based plain ‘chicken’ contained significantly lower sugar than ‘standard’ meat-based equivalents. However, only pre-cooked chicken products were included for comparability with the equivalent PBMs, and these products may include additional carbohydrates such as dextrose and starch. Typically, higher sugar content in PBM could be a health concern. However, Gallani and Klapp^(82)^ suggest the relatively small amounts involved could be managed within a balanced diet. Nevertheless, manufacturers should consider novel processing methods to reduce the overall sugar content.

Notably, PBM products demonstrated a relatively higher dietary fibre content across all categories which concurs with previous studies within the UK and across Europe, Australia, Asia and South Africa.^(35,36,39,40,42,46,68)^ The current fibre consumption of UK adults is substantially below the 30 g daily recommendation^(83)^ and, considering the associated health benefits of high fibre diets,^(84)^ displacing meat with PBM may have significant health benefits.

Consumers are often sceptical whether the content of protein in PBM is comparable to meat products as a protein source.^(85–87)^ Our findings support these concerns and previous studies,^(85–87)^ with significantly lower protein in PBM versus most ‘standard’ and ‘reduced’ meat-based product categories. Current UK adult consumption often exceeds recommended daily protein requirements.^(88)^ Therefore, although lower in protein, PBM products with higher dietary fibre and lower saturated fat content could still facilitate adherence to other dietary recommendations. However, our study did not consider amino acid profile, bioavailability and bio-accessibility. Thus, further work is warranted to compare the protein quality between PBM and their meat comparators.

Significantly higher salt in PBM versus ‘standard’ meat-based products across most categories contradicts previous work in Australia^(43,48)^ and Asia^(45,69)^ which demonstrated PBM products were generally more favourable than conventional meat. However, other studies in the UK and across Europe reported similar observations.^(34,38,39,46)^ Increased salt consumption is associated with poor cardiovascular health and diet-related deaths.^(89–91)^ Hence published recommendations propose salt reduction targets with specific guidance for meat alternative products.^(92)^ For example, limiting salt content per 100 g of product to 0.63 g (plain meat e.g. mince), 1.19 g (meat-free e.g. burgers) and 1.78 g (meat-free bacon). Only in plant-based burgers, breaded/battered ‘chicken’ and meatball product categories was median salt content within these thresholds which suggests further work is required to reduce the salt content of PBM. The difference between PBM and ‘reduced’ meat-based comparators was more nuanced with only plant-based burger and mince alternatives being significantly higher. Ruusunen and Puolanne^(93)^ reported lower perceived saltiness in lean versus regular meat products, suggesting that ‘reduced’ meat products have added salt to replicate ‘standard’ meat sensorial profiles. Our findings suggest that manufacturers must prioritise salt reduction in PBM products. This is particularly important given that the current consumption level of salt in the UK is reportedly ∼40 % higher than the recommended daily maximum of 6 g.^(92)^ Possible suggestions include ingredients such as herbs and spices^(94)^ or potassium chloride^(95)^ to reduce salt content without compromising the sensorial quality.

## Nutrient profiling model

An overview of nutritional profiles alongside nutritional composition in this study improved understanding regarding the nutritional quality and healthiness of PBM products which may support ‘healthier’ consumers choices. In line with previous work,^(39,40,47)^ PBM products had significantly lower (‘healthier’) NPM scores compared to ‘standard’ meat-based equivalents across all product categories. ‘Reduced’ meat-based equivalents also scored significantly lower (‘healthier’) against ‘standard’ meat products but there was no significant difference in comparing ‘reduced’ meat with PBM across all product categories. However, a greater proportion of PBM products were classified as ‘healthier’ compared to both ‘standard’ and ‘reduced’ meat-based equivalents. This reflects previous findings that PBM products were more likely to be classified in ‘healthier’ profiling categories.^(41,42,44)^ Hence, while ‘reduced’ meat could offer ‘healthier’ alternatives to ‘standard’ meat products, there may be incremental health benefits when adopting PBM. However, NPM does not consider other key dimensions of health including micronutrient content and degree of processing.

## Degree of processing

Applying the NOVA classification system for degree of processing, we found that all PBM, all-but one ‘reduced’ meat product category and most ‘standard’ meat-based products were characterised as UPF. This is an important contribution to the current debate regarding the association between UPF and adverse health outcomes^(96)^ as, despite being categorised as UPF, a larger proportion of PBM products were characterised as ‘healthier’ than meat-based equivalents according to the UK NPM. These results are consistent with a limited number of studies which have also combined nutritional profiling and degree of processing within Spanish,^(46)^ Australian^(48)^ and Canadian^(47)^ PBM markets, emphasising that not all ultra-processed products are inherently unhealthy.^(51,53,96)^ While these results again suggest that PBM may offer a ‘healthier’ steppingstone to reducing meat consumption in a well-balanced diet, the NPM fails to consider the impact of the various ultra-processing mechanisms, beyond nutritional composition, on health outcomes. The recent Lancet series on UPF and human health purports factors such as hyper-palatability, disrupted food matrices, and increased exposure to toxic compounds may contribute to the deleterious health outcomes associated with increased consumption of UPFs.^(97)^ Thus, further research is warranted to further understand the potential health implications of ultra-processing mechanisms independent of nutritional composition.

## Micronutrients

As with previous studies,^(29,38,40,44,69,98)^ micronutrient data was limited to a small proportion of PBM products but demonstrated variability in iron, zinc and vitamin B_12_ content. In comparison to reference meat data, PBM products appear to have relatively higher levels of zinc and iron and contribute a greater proportion to UK adult DRVs. Meanwhile, vitamin B_12_ content was typically lower and contributed less to the DRVs. However, vitamin B_12_ is exclusive to animal sources and vitamin B_12_ in PBM is reliant on fortification.^(99)^ PBM products contribution to DRVs may also be impacted by lower bioaccessability and bioavailability of micronutrients^(99,100)^ and the presence of antinutrients (e.g. tannins) may further influence absorption rates.^(101)^ However, restricted on-pack information indicates that laboratory compositional analyses may be needed to better evaluate PBM micronutrient profiles. Standards of fortification for PBM, across all product categories, may address possible nutritional deficiencies and consideration should be given to the impact of processing on the food matrix, and the digestibility and absorption of micronutrients.

## Product claims

Health and nutritional claims support consumers to make healthier dietary choices.^(16)^ Across all product categories, only one PBM and few ‘standard’ and ‘reduced’ meat-based products made health-related claims while a notably higher proportion of PBM products made at least one nutritional claim, primarily related to protein, saturated fat or dietary fibre content. Previous authors have also reported protein-related claims on a large proportion of PBM products,^(38,40–42)^ which may be a response to previously mentioned consumer scepticism^(85–87)^ or the current media attention surrounding high protein products.^(102)^ Surprisingly, although numerous products met European Commission^(58)^ eligibility criteria for nutritional claims, many did not include on-pack information. Hence, PBM manufacturers should consider on-pack nutritional claims to promote them as part of healthier, more sustainable diets. This may enhance the perception of PBM products as healthy in addition to the more frequent non-health claim statements (e.g. ‘vegan’ and ‘plant-based’).^(20)^

## Product cost

PBM products were significantly more expensive than ‘standard’ meat-based equivalents across four categories. This increased cost may relate to a range of factors including processing costs to simulate nutritional and organoleptic qualities of meat and a lower market share.^(103,104)^ Agricultural subsidies that promote intensive production of low-cost meat products alongside a relative paucity of subsidies for PBM may also play a role in the lack of price parity.^(105)^ Previous authors of studies within the UK and across Europe, Asia and South Africa have also raised concern regarding affordability,^(29,34,37)^ particularly considering that cost is a key factor influencing consumer adoption of PBM products.^(10,99,106)^ Novel strategies to reduce production costs and policy interventions such as PBM subsidies and the implementation of a meat tax may strengthen the market competitiveness of PBM while simultaneously creating a financial barrier to meat purchasing behaviour.^(106,107)^ In the other four product categories, there was a lack of significant difference between PBM and ‘standard’ meat-based equivalents and high level of inter-category variation between PBM and ‘reduced’ meat-based products. Recent evidence from non-UK markets suggests the gap between PBM and meat-based products is ameliorating.^(47,108)^ While recent inflation pressures and supply chain disruption may have also impacted on our findings.^(47,109)^ It should be noted that meat-based products designed as ‘healthier’ options are also more expensive versus ‘standard’ meat-based products.^(110)^ Hence a longitudinal study design may improve understanding regarding the relative affordability of PBM over time.

## Study limitations

Our study had several limitations that should be acknowledged. Firstly, we retrieved nutritional information from on-pack labelling which limited our understanding of non-mandatory nutrients including vitamins and minerals. Further work, using laboratory nutritional analyses, is warranted to improve knowledge regarding the micronutrient content of PBM, particularly given that meat is a rich source of zinc, iron and vitamin B_12_. It is also important to note that nutritional labelling fails to consider the influence of the food matrix on nutritional and health outcomes.^(111)^ Thus future studies are required to understand the impact of PBMs food matrices on the digestion and absorption of nutrients in addition to their mechanistic qualities. This is particularly important considering food processing methods influence the food matrix^(112)^ and all PBM products were identified as ultra-processed. Finally, while the current study targeted dominant UK supermarkets, representing ∼97% of the UK market share, it is likely that some products may not have been identified. While future studies could consider including supplementary in-store audits, careful consideration must be given to scalability due to the potential influence of store size, regional variation, consumer purchasing habits and supply chain issues on UK-wide representativeness.

## Conclusion

We report considerable variation in nutritional composition and affordability of PBM when compared against meat-based equivalents in the current UK market. PBM products were typically more favourable than ‘standard’ meat-based equivalents for energy density, dietary fibre, total and saturated fat content. However, they contain significantly higher salt in the majority of categories and fortification strategies may be needed to ensure sufficient micronutrient content. Future product development also needs to balance affordability and sensorial quality of PBM. The comparison between PBM and ‘reduced’ meat-based product categories was more nuanced and will require further investigation.

All PBM and the vast majority of meat products were characterised as UPF, yet PBM demonstrated lower (‘healthier’) NPM scores versus ‘standard’ meat-based equivalents across all product categories. Although no statistically significant difference was found between PBM and ‘reduced’ meat equivalents, a higher proportion of PBM products were classified as ‘healthier’. This suggests additional health benefits when adopting PBM and a subsequent need to improve consumer literacy regarding nutritional labelling and focus on the ‘healthiness’ of PBM products. For example, that not all ultra-processed foods are inherently ‘unhealthy’. Although minimally processed, ‘traditional’ plant-based whole foods should be prioritised, PBM may offer a steppingstone in the transition to reduce meat consumption and adopting more plant-rich diets. Further research will be required to understand the impact of processing on PBM associated health outcomes and inform future dietary guidance.

## Supporting information

Flint et al. supplementary material 1 Flint et al. supplementary material

Flint et al. supplementary material 2 Flint et al. supplementary material

Flint et al. supplementary material 3 Flint et al. supplementary material

Flint et al. supplementary material 4 Flint et al. supplementary material

## Supplementary material

The supplementary material for this article can be found at https://doi.org/10.1017/jns.2026.10083
