# Supplementary material for: ‘Do plant-based meats offer a steppingstone towards healthier choices? A cross-sectional audit of the UK market’
Source: J Nutr Sci. 2026 Mar 27;15:e20. doi: 10.1017/jns.2026.10083 (PMC13126074; doi:10.1017/jns.2026.10083)
Supplement: Flint et al. supplementary material 1 — Flint et al. supplementary material [file S2048679026100834sup001.docx]

Inclusion:

- Plant-based meat products designed to mimic the sensorial qualities and to be handled and cooked in the same manner as meat-based equivalents, enabling direct substitution in familiar consumption contexts (e.g., plant-based sausages and plant-based mince).

Exclusion:

- Any food or food products derived from plants which are not designed to imitate meat-based products. Examples include whole foods (e.g., fruit and vegetables) and commercially available products (e.g., bean burgers, dairy alternatives, tofu, tempeh, and falafel).
- Non terrestrial meat alternative products e.g., plant-based alternatives to fish and seafood products
- Products with any additional component (e.g., sauce/kiev) to enable a direct and standardised comparison between the plant-and meat-based products and to minimise risk of potential confounding from added components.

Defined search terms included “plant-based” “meat-free” “meat alternative” “meat substitute” “vegetarian” and “vegan” (Table S1). The same approach was used to identify equivalent meat-based products although defined meat-based search terms were adopted to identify suitable comparators.

Supplementary Table 1: Plant-based meat product classification criteria.

| **Product Category** | **Description** |
| --- | --- |
| ‘Beef’ Burgers | Plant-based products mimicking “Burgers” “Patties/Patty” “Quarter Pounders” |
| ‘Pork’ Sausage | Plant-based products mimicking “Sausages” “Hot Dogs” “Bangers” |
| Breaded/ Battered ‘Chicken’ | Plant-based products mimicking breaded/battered chicken “Nuggets”” “Chick’nNuggets” “Chick’N” “Chick’N Pieces” “Dippers” “Goujons” “Southern-fried” “Escalopes” “Schnitzel” |
| Plain ‘Chicken’ | Plant-based products mimicking chicken “Strips” “Bites” “Chunks” “Pieces” “Chargrilled” “Breast” “Fillets” |
| ‘Beef’ Meatballs | Plant-based products mimicking “Meatballs” and “Balls” |
| ‘Beef’ Mince | Plant-based products mimicking “Mince” |
| Bacon | Plant-based products mimicking “Bacon” and “Rashers” |
| Deli Meat | Plant-based products mimicking “pastrami” “ham slices” “chicken slices” “salami” “pepperoni” “chorizo” |
