# Supplementary material for: ‘Do plant-based meats offer a steppingstone towards healthier choices? A cross-sectional audit of the UK market’
Source: J Nutr Sci. 2026 Mar 27;15:e20. doi: 10.1017/jns.2026.10083 (PMC13126074; doi:10.1017/jns.2026.10083)
Supplement: Flint et al. supplementary material 2 — Flint et al. supplementary material [file S2048679026100834sup002.docx]

**Supplementary Table 2: List of ingredients used as markers of ultra-processed foods^(54-55)^**

| Acesulfame K | High-fructose corn syrup |
| --- | --- |
| Advantame | Humectant |
| Agar | Hydrogenated/interesterified oil |
| Agent (e.g., anti-caking, bulking, carbonating, foaming, de-foaming, firming, gelling, glazing, leavening, raising) | Hydrolysed protein |
| Anti-bulking | Improver |
| Aspartame | Inosinic acid |
| Barley malt extract | Inositol |
| Beta carotene | Insoluble fibre |
| Binder | Interesterified oil |
| Caffeine | Invert sugar |
| Calcium 5'-ribonucleotides | Inverted refiners syrup# |
| Calcium diglutamate | Inverted sugar syrup# |
| Calcium guanylate | Isolated soy protein |
| Calcium inosinate | Isomalt |
| Carbon dioxide | Lactitol |
| Carrageenan | Lactose |
| Casein | Magnesium diglutamate |
| Charcoal | Maltitol |
| Coffee essence | Maltitol syrup |
| Colour stabiliser | Maltodextrin |
| Colour/Color | Maltol |
| Confectioners glaze | Maltose |
| Corn syrup (incl. high fructose) | Mannitol |
| Cyclamate | Mechanically separated meat |
| Cyclamic acid | Menthol |
| Dextrin | Modified (maize/potato) starch |
| Dextrose | Monoammonium glutamate |
| Dipotassium guanylate | Monoglyceride |
| Dipotassium inosinate | Monopotassium glutamate |
| Disodium 5'-ribonucleotides | Monosodium glutamate |
| Disodium guanosinemonophosphate | Neohesperidine DC |
| Disodium guanylate | Neotame |
| Disodium inosinate | Non-culinary ingredients |
| Dried whey | Oligosaccharides |
| Dye | Pectin |
| E420 | Polydextrose |
| E421 | Polyglycitol |
| E620 – E629 | Propane |
| E630 – E637 | Propellant |
| E640 | Protein powder/isolate (including hydrolysed/hydrolyzed) |
| E650 | Saccharin |
| E950 – E959 | Salt of aspartame-acesulfame |
| E960 – E969 | Sequestrant |
| Emulsifier | Sodium guanylate |
| Emulsifying salt | Sodium nitrite/ Sodium nitrate |
| Erythritol | Soluble fibre |
| Esters | Sorbitol |
| Ethyl maltol | Sorbitol syrup |
| Extract | Soy lecithin |
| Fibre | Soy protein isolate |
| Fibre isolate | Stabiliser/stabilizer |
| Flavour enhancer | Steviol glycoside |
| Flavour/Flavor | Sucralose |
| Food additive (including processing aid, sequestrants 452, 385, etc.) | Sucrose fatty acid esters |
| Fructose | Sweetener (including acesulphame potassium (950) and aspartame (951) etc.) |
| Glucose-fructose syrup | Thaumatin |
| Fructose-glucose syrup# | Thickener |
| Fruit juice concentrate | Trehalose |
| Glucose | Triglycerides |
| Glucuronolactone | Whey derivatives |
| Glutamic acid | Whey permeate |
| Gluten | Whey powder |
| Glycerine | Whey protein |
| Glycerol | Whey solids |
| Glycine and its sodium salt | Whey |
| Guanylic acid | Xylitol |
| Gums (Arabic, guar, vegetable, xanthan) | Zinc acetate |
