# Supplementary material for: ‘Do plant-based meats offer a steppingstone towards healthier choices? A cross-sectional audit of the UK market’
Source: J Nutr Sci. 2026 Mar 27;15:e20. doi: 10.1017/jns.2026.10083 (PMC13126074; doi:10.1017/jns.2026.10083)
Supplement: Flint et al. supplementary material 3 — Flint et al. supplementary material [file S2048679026100834sup003.docx]

**Supplementary Table 3: Proportion (number and %) of plant-based meat (PB) and standard (MB) and ‘reduced’ (RMB) meat-based equivalent products eligible to make a nutritional claim according to the European Commission (2012) criteria across each product category.**

|  | **Nutritional Claims** | | | | | | | |
| --- | --- | --- | --- | --- | --- | --- | --- | --- |
|  | **Protein** | | **Fibre** | | **Sugar** | | **Low Fat** | **Low**  **Saturated Fat** |
|  | **Source of** | **High in** | **Source of** | **High in** | **Low** | **Sugar Free** |  |  |
| **PB Burger** | 32  (100.0) | 29  (90.6) | 26  (81.3) | 10  (31.3) | 32  (100.0) | 9  (28.1) | 1  (3.1) | 9  (28.1) |
| **MB Burger** | 98  (100.0) | 97  (99.0) | 0  (0.0) | 0  (0.0) | 96  (98.0) | 63  (64.3) | 0  (0.0) | 0  (0.0) |
| **RMB Burger** | 12  (100.0) | 12  (100.0) | 0  (0.0) | 0  (0.0) | 12  (100.0) | 6  (50.0) | 3  (25.0) | 4  (33.3) |
| **PB Sausage** | 30  (100.0) | 28  (93.3) | 25  (83.3) | 7  (23.3) | 30  (100.0) | 8  (26.7) | 0  (0.0) | 14  (46.7) |
| **MB Sausage** | 233  (99.1) | 168  (71.5) | 4  (1.7) | 1  (.4) | 223  (94.9) | 57  (24.3) | 0  (0.0) | 2  (.9) |
| **RMB Sausage** | 12  (100.0) | 12  (100.0) | 2  (16.7) | 0  (0.0) | 12  (100.0) | 3  (25.0) | 2  (16.7) | 3  (25.0) |
| **PB Breaded/Battered ‘Chicken’** | 32  (91.4) | 12  (34.3) | 32  (91.4) | 15  (42.9) | 35  (100.0) | 9  (25.7) | 0  (0.0) | 21  (60.0) |
| **MB Breaded/Battered Chicken** | 389  (99.7) | 344  (88.2) | 7  (1.8) | 0  (0.0) | 388  (99.5) | 112  (28.7) | 0  (0.0) | 178  (45.6) |
| **RMB Breaded/Battered Chicken** | - | - | - | - | - | - | - | - |
| **PB Plain ‘Chicken’** | 44  (100.0) | 2  (4.5) | 40  (90.9) | 16  (36.4) | 3  (6.8) | 25  (56.8) | 2  (4.5) | 38  (86.4) |
| **MB Plain Chicken** | 161  (100.0) | 0  (0.0) | 2  (1.2) | 0  (0.0) | 11  (6.8) | 59  (36.6) | 78  (48.4) | 97  (60.2) |
| **RMB Plain Chicken** | - | - | - | - | - | - | - | - |
| **PB Meatballs** | 17  (100.0) | 17  (100.0) | 14  (82.4) | 6  (35.3) | 17  (100.0) | 1  (5.9) | 0  (0.0) | 10  (58.8) |
| **MB Meatballs** | 37  (100.0) | 35  (94.6) | 0  (0.0) | 0  (0.0) | 37  (100.0) | 32  (86.5) | 0  (0.0) | 0  (0.0) |
| **RMB Meatballs** | 11  (100.0) | 11  (100.0) | 0  (0.0) | 0  (0.0) | 11  (100.0) | 5  (45.5) | 3  (27.3) | 5  (45.5) |
| **PB Mince** | 18  (100.0) | 18  (100.0) | 14  (77.8) | 3  (16.7) | 18  (100.0) | 3  (16.7) | 4  (22.2) | 14  (77.8) |
| **MB Mince** | 56  (100.0) | 56  (100.0) | 0  (0.0) | 0  (0.0) | 56  (100.0) | 56  (100.0) | 0  (0.0) | 0  (0.0) |
| **RMB Mince** | 32  (100.0) | 32  (100.0) | 0  (0.0) | 0  (0.0) | 32  (100.0) | 32  (100.0) | 3  (9.4) | 1  (3.1) |
| **PB Bacon** | 10  (100.0) | 10  (100.0) | 9  (90.0) | 2  (20.0) | 10  (100.0) | 0  (0.0) | 3  (30.0) | 9  (90.0) |
| **MB Bacon** | 268  (100.0) | 264  (98.5) | 0  (0.0) | 0  (0.0) | 268  (100.0) | 214  (79.9) | 5  (1.9) | 12  (4.5) |
| **RMB Bacon** | 21  (100.0) | 21  (100.0) | 0  (0.0) | 0  (0.0) | 21  (100.0) | 12  (57.1) | 2  (9.5) | 6  (28.6.) |
| **PB Deli Meat** | 23  (100.0) | 21  (91.3) | 18  (78.3) | 6  (26.1) | 23  (100.0) | 4  (17.4) | 4  (17.4) | 20  (87.0) |
| **MB Deli Meat** | 893  (99.4) | 823  (91.6) | 2  (.2) | 1  (.1) | 896  (99.8) | 436  (48.6) | 303  (33.7) | 392  (43.7) |
| **RMB Deli Meat** | 12  (100.0) | 12  (100.0) | 0  (0.0) | 0  (0.0) | 12  (100.0) | 4  (33.3) | 7  (58.3) | 8  (66.7) |
| **Total PB** | 206  (98.6) | 137  (65.6) | 178  (85.2) | 65  (31.1) | 168  (80.4) | 59  (28.2) | 14  (6.7) | 135  (64.6) |
| **Total MB** | 2135  (99.6) | 1787  (83.4) | 15  (.7) | 2  (.1) | 1975  (92.2) | 1029  (48.0) | 386  (18.0) | 681  (31.8) |
| **Total RMB** | 100  (100.0) | 100  (100.0) | 2  (2.0) | 0  (0.0) | 100  (100.0) | 62  (62.0) | 20  (20.0) | 27  (27.0) |
