# Supplementary material for: ‘Do plant-based meats offer a steppingstone towards healthier choices? A cross-sectional audit of the UK market’
Source: J Nutr Sci. 2026 Mar 27;15:e20. doi: 10.1017/jns.2026.10083 (PMC13126074; doi:10.1017/jns.2026.10083)
Supplement: Flint et al. supplementary material 4 — Flint et al. supplementary material [file S2048679026100834sup004.docx]

**Supplementary Table 4:** **Prevalence (number and %) of additional statements present on plant-based meat, standard and ‘reduced’ meat-based products across each product category.**

| **Additional Statements** | **Total** | | **PB** | | **MB** | | **RMB** | |
| --- | --- | --- | --- | --- | --- | --- | --- | --- |
|  | **N** | **%** | **N** | **%** | **N** | **%** | **N** | **%** |
| Gluten Free | 369 | 15.0 | 18 | 8.6 | 326 | 15.2 | 25 | 25.0 |
| No Artificial Colours, Flavours, Hydrogenated Fats, Preservatives, Additives/ Ingredients; Naturally Delicious Colours & Flavours/ Natural Colours and Flavours. | 315 | 12.8 | 23 | 11.0 | 285 | 13.3 | 7 | 7.0 |
| Plant-Based/ Suitable for Vegans/ Suitable for Vegetarians/ Meat-Free | 202 | 8.2 | 202 | 96.7 | 0 | 0.0 | 0 | 0.0 |
| Organic | 44 | 1.8 | 0 | 0.0 | 40 | 1.9 | 4 | 4.0 |
| Made without Nitrite/Nitrites/ Nitrite Free/ Made without Added Nitrites | 35 | 1.4 | 0 | 0.0 | 35 | 1.6 | 0 | 0.0 |
| Generic Health/Nutrition Claim e.g., Live Better/Nutritionist Approved etc | 35 | 1.4 | 11 | 5.3 | 19 | .9 | 5 | 5.0 |
| GMO Free/ Free-From Genetically Modified Ingredients | 30 | 1.2 | 15 | 7.2 | 13 | .6 | 2 | 2.0 |
| Free-Range | 30 | 1.2 | 0 | 0.0 | 30 | 1.4 | 0 | 0.0 |
| Lactose Free | 24 | 1.0 | 0 | 0.0 | 24 | 1.1 | 0 | 0.0 |
| No Soy/ Soya Free | 17 | .7 | 14 | 6.7 | 3 | .1 | 0 | 0.0 |
| Dairy Free | 13 | .5 | 2 | 1.0 | 11 | .5 | 0 | 0.0 |
| Milk Free | 10 | .4 | 0 | 0.0 | 10 | .5 | 0 | 0.0 |
| No Additives/ Free-From Additives/ No Preservatives | 8 | .3 | 3 | 1.4 | 5 | .2 | 0 | 0.0 |
| Cholesterol Free | 7 | .3 | 7 | 3.3 | 0 | 0.0 | 0 | 0.0 |
| Nutri-Score Label | 6 | .2 | 0 | 0.0 | 6 | .3 | 0 | 0.0 |
| 0% Palm Oil/ Palm Oil Free | 5 | .2 | 5 | 2.4 | 0 | 0.0 | 0 | 0.0 |
| No Added Glutamates/ No MSG | 4 | .2 | 0 | 0.0 | 4 | .2 | 0 | 0.0 |
| No Added Colours | 3 | .1 | 0 | 0.0 | 3 | .1 | 0 | 0.0 |
| Wheat Free | 2 | .1 | 0 | 0.0 | 2 | .1 | 0 | 0.0 |
| No Added Phosphates | 2 | .1 | 0 | 0.0 | 2 | .1 | 0 | 0.0 |
| Allergen Free | 2 | .1 | 0 | 0.0 | 2 | .1 | 0 | 0.0 |
